# The past, present, and future of tumour deposits in colorectal cancer: Advancing staging for improved prognosis and treatment decision‐making

**DOI:** 10.1111/jcmm.18562

**Published:** 2024-08-27

**Authors:** Zhengyan Chang, Huijun Fu, Jiaqi Song, Cheng Kong, Ruting Xie, Man Pi, Xuechen Sun, Wentao Zhang, Yifan Liu, Ruizhi Huang, Tingsong Yang, Dongyan Han

**Affiliations:** ^1^ Department of Pathology, Shanghai Tenth People's Hospital Tongji University School of Medicine Shanghai China; ^2^ Shanghai Jiao Tong University School of Medicine Shanghai China; ^3^ Department of Colorectal Surgery Fudan University Shanghai Cancer Center Shanghai China; ^4^ Department of Urology, Shanghai Tenth People's Hospital Tongji University School of Medicine Shanghai China; ^5^ Department of Burn Surgery The First Affiliated Hospital of Naval Medical University Shanghai China; ^6^ Department of General Surgery, Shanghai Tenth Peoples' Hospital Tongji University School of Medicine Shanghai China

**Keywords:** AJCC, bibliometric analysis, colorectal cancer, TNM staging system, tumour deposits

## Abstract

Tumour deposits (TDs) significantly impact colorectal cancer (CRC) prognosis. Integrating TDs into the TNM staging system can enhance individualized disease management. Keeping abreast of evolving TDs research is pivotal for clinical advancement. We comprehensively reviewed both recent and popular literature to grasp the field's essence. Subsequently, a data retrieval sourced articles on TDs in CRC for bibliometric analysis, spanning from 1 January 1935 to 30 April 2023. Bibliometrix software facilitated paper analysis and visualization. Bibliometric indicators, the trends and hotspots were determined. A total of 2147 articles were successfully retrieved. Brown G emerged as the most productive author, and the USA as the most prolific country. Central South University and Memorial Sloan Kettering Cancer Center led productivity. Bradford's law categorized 48 journals into zone 1. Keywords co‐occurrence analysis identified three main clusters: the application of TDs in TNM staging, the pathogenesis of TDs, and the assessment of TDs. The trend topic analysis highlighted research focused on refining TDs incorporation into tumour staging. TDs wield enduring medical significance, shaping ongoing research. Much literature focused on confirming TD's prognostic value and optimizing TNM integration. Additionally, it is worth highlighting that TD's enigmatic pathogenesis demands research priority, as it holds the potential to unveil concealed knowledge regarding their development.

## 1 INTRODUCTION

Colorectal cancer (CRC) was the second most common cause of cancer related death and ranked third with regard to incidence.^1^ Notably, it was increasingly regarded as one of the most prominent indicators of the cancer in nations experiencing rapid sociological and economic changes.^2^

Previously, many markers were found to be prognostic in CRC, including perineural invasion, vascular emboli and tumour deposits (TDs).^3^ TDs were first reported as a kind of vascular invasion in 1935.^4^ They were characterized as tumour nodules outside of lymph nodes which were originally identified in the pericolic adipose tissue and were also found in perirectal, or adjacent mesenteric adipose tissue surrounding the primary colorectal lesion later.^5^ The frequency of TDs reported in CRC ranged from 4.9% to 41.8%, the average frequency was 22%^6^ and its vital role in prognosis has been under extensive study. For example, in a meta‐analysis with 10,106 CRC patients, TDs was found to be an important indicator for disease‐free survival and overall survival.^6^ Due to its important role in prognosis,^7^, ^8^, ^9^ an integration of it into traditional TNM staging system has been a topic but well acknowledge agreement was still not reached.

Historically, TDs were first adopted in the 5th edition of TNM staging system in 1997 and the definition of it continuously evolved with the update of the TNM staging system.^10^ The 5th and 6th editions of TNM staging system adopted the “size principle” and “contour principle,” respectively, and were both refuted later.^11^, ^12^ The 7th edition excluded replaced lymph from TDs and tailored the “N1c” category for TDs in the absence of lymph node metastasis (LNM).^5^, ^13^ In the 8th edition, lesion with obvious remaining lymph node, vascular, or neural structures was ruled out.^3^, ^14^ However, the 7th and 8th edition were both blamed for uncontrollable intra‐observer variability.^15^ Also, the present N1c category still ignored the varied origins and the number of TDs, which might prevent TDs from operating at full capacity.^6^ Furthermore, the rationality of the latest TDs criteria was questioned.^16^

Based on this background, the role and status of TDs in tumour staging remains controversial at present. As a result, it is necessary to sum up related researches to guide clinical practice. Thus, our study aimed to provide a timely review of current researches in this field. To provide a more comprehensive and complete analysis of the existing literature, a bibliometric analysis coupled with manual review was carried out. The bibliometric analysis was adopted to carry out a quantitative analysis of collected literatures and identify future research hotspots. Based on the information provided by bibliometric analysis, a further manual review was carried out. Bibliometric researches are mathematical and statistical analyses of qualified literatures published on an interested topic during a set time period to gain a grasp of the past development, status quo and future direction of a certain field.^17^ This practical analytical strategy has so far been used in several medical fields, such as rheumatology,^17^ microbiology^18^ and neurology.^19^ Bibliometric analyses have long been applied in medicine and have been popular among medical researches due to the ability of bibliometric analyses to provide comprehensive, quantitative research and the increasing demand for evidence‐based research in medical fields.^20^ As a result, the bibliometric study was selected for this study. As for the literature database, the Web of Science was chosen for being the largest database of academic materials, which improves the accuracy of our evaluation of influence.^17^ Up to now, there still has been a dearth of bibliometric study in the area of tumour deposition in CRC. To this end, we conducted a bibliometric analysis as a supplementary study of this review, aiming to get a more comprehensive understanding of the achieved research output, predict the future hot topics and guide the future researches in this field.

Our research mainly focused on solving the following six research questions:

1. What is the current state of research on TDs in CRC, and how has it evolved over time?

2. What is the clinical significance and prognostic value of TDs, and how do they compare to traditional LNMs?

3. How have TDs been integrated into the TNM staging system, and what challenges exist in their classification and staging?

4. Who are the key researchers contributing to the literature on TDs in CRC?

5. What are the institutions and countries contributing the most to the literature on TDs in CRC?

6. What are the emerging research hotspots, trends, and gaps in the field of TDs in CRC, and how can they guide future research efforts?

## 2 METHODS

### 2.1 Data source and retrieval strategies

After reviewing part of the popular literature and the latest literature to form a preliminary framework of this field, a comprehensive data retrieval was carried out. Web of Science Core Collection was used to search for papers on the TDs in intestinal cancer. The retrieval strategy was ((TS = intestinal cancer) OR (TS = colonic carcinoma) OR (TS = colonic cancer) OR (TS = rectal cancer) OR (TS = rectal carcinoma) OR (TS = colon carcinoma) OR (TS = colon cancer)) AND ((TS = TD) OR (TS = tumour deposition) OR (TS = TD) OR (TS = cancer deposit) OR (TS = carcinoma deposit) OR (TS = tumour nodule) OR (TS = cancer nodule) OR (TS = carcinoma nodule)). The literature searches and data extractions were completed on 30 April 2023 in order to prevent divergence from the database's periodic updates. Only articles and reviews were included in the study and certain publications that were not related to the theme were excluded by careful examination of the title and abstract. At last, a total of 2147 articles were included with all data exported in plain text format. The detailed bibliographic information, including title, authors, publication year, country, institution, keywords, citations, abstract and reference, was converted to R data frame.

### 2.2 Methodology

The analysis was programmed in R to be flexible and facilitate integration with other statistical and visual applications.^21^

Basic descriptive analyses, including annual analysis of publications, the most cited references, documents, journals and the most productive countries, institutions, authors, were performed by the bibliometrix package in R version 4.2.0 (Institute for Statistics and Mathematics, Vienna, Austria; www.r‐project.org).^21^ Lotka's law was adopted to demonstrate the publication frequency of authors.^22^ According to Lotka's law, the productivity of any individual author (*x*) is inversely related to the number of writers, each of whom is credited with *x* papers.^23^

The H‐index was also utilized as a metric for the volume and impact of journals' and scholars' scientific output.^24^ For each researcher, it signified that h of his documents had at least h citations, whereas the remaining documents had fewer citations than h.^25^ The h‐index might be more helpful in the analysis of academic output than other citation indexes because it took into account both quantitative and qualitative components of the output.^26^ The use of Lotka's law and h‐index could reveal the most influential author in this field, helping the readers to keep updated with the latest discoveries in CRC and following the benchmark in this field.

As for determining core sources, Bradford's law was used.^21^ In 1948, Bradford's law, an empirical rule of scientific journal literature, was put forward. Bradford's law sorted out a core of periodicals that were more focused on the given subject by placing the scientific journals in declining order based on the productivity of articles on a certain subject. The following groups of journals that published the same number of articles as the nucleus were then separated into zones, in which the numbers of periodicals in the nucleus and succeeding zones followed 1: n: n^2^:…^27^ By revealing the most productive and influential periodicals, the readers can read the latest and best quality research in CRC in a more targeted manner.

To show the progress of the topic over time and the research hotspots, co‐word analysis was conducted using the bibliometrix package in R version 4.2.0 (Institute for Statistics and Mathematics, Vienna, Austria; www.r‐project.org).^21^ Based on the acquired data, bibliometrix could successfully carry out a science mapping analysis thoroughly. Using a word co‐occurrence network, keyword co‐occurrence analysis was able to map and cluster terms gathered from keywords, titles or abstracts in a bibliographic collection. The simultaneous occurrence of two keywords in a publication was referred to as co‐word/co‐occurrence in a semantic relationship.^28^ The correlation between two terms increases as their co‐occurrence frequency increases. Keyword co‐occurrence analysis was a crucial technique for analysing topics and summarizing research hotspots. Therefore, keyword co‐occurrence analysis could illustrate many subfields and their connections in order to spot emerging trends.

## 3 RESULTS

### 3.1 Annual analysis of publication

**FIGURE 1:**
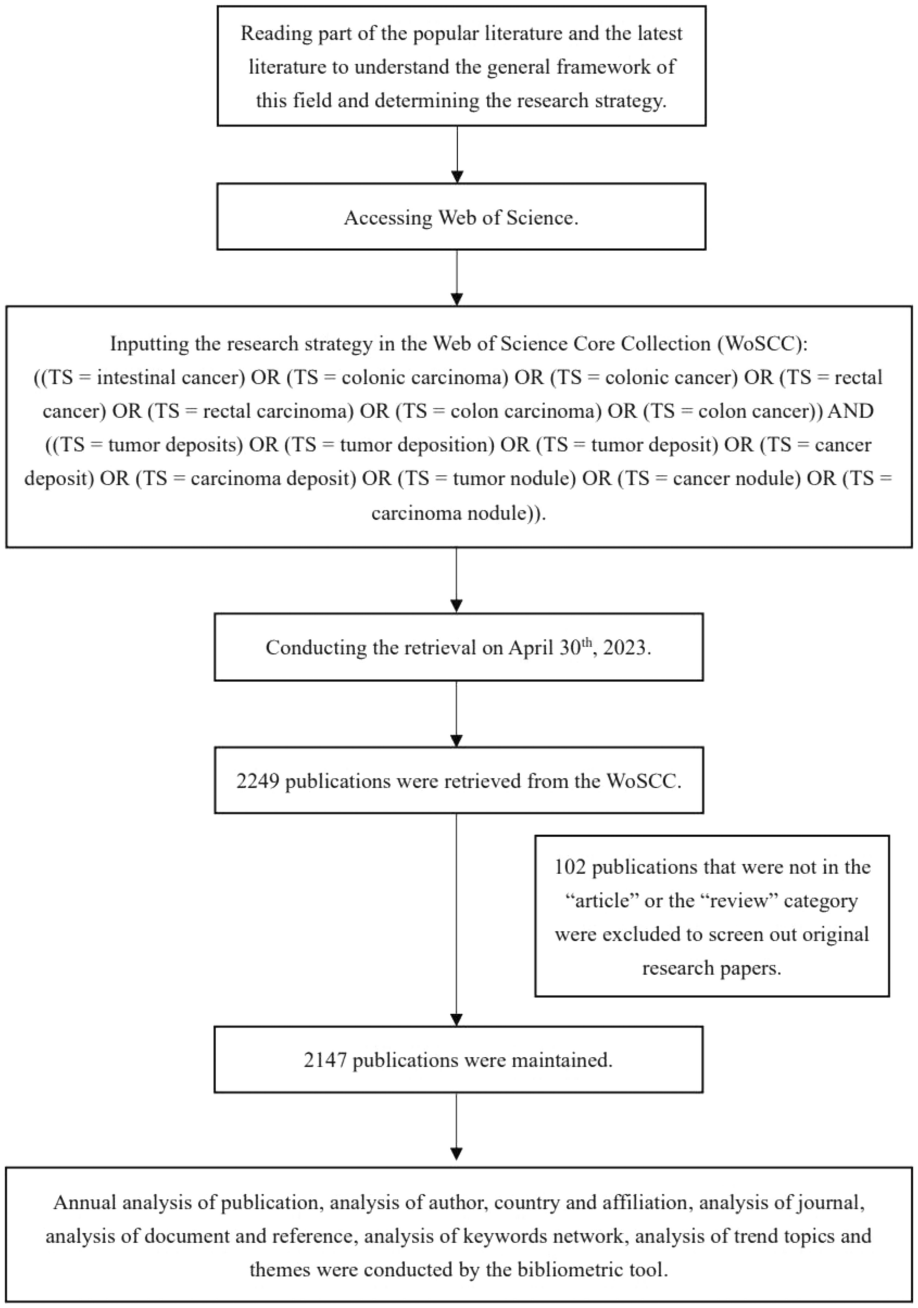
Flowchart of the research.

**FIGURE 2:**
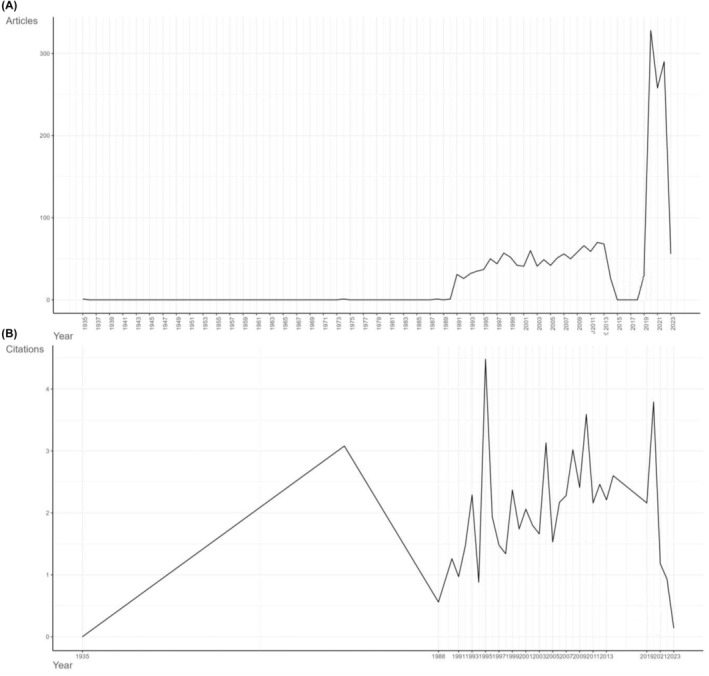
Annual publications and citations. (A) Annual publications of TDs in CRC from 1900 to 2023. (B) Annual average of citations to articles between 1900 and 2023.

After conducting the process in Figure 1, a total of 2147 articles were retrieved from 1900 to 2023, cited 63,340 times all together (Table 1). The publication number and citation number of each year could indicate the popularity and the speed of development. Figure 2A demonstrates the annual scientific production from 1935 to 2023. Before 1990, there was few scientific output in this field. Publication began to rise after 1990 but the number remained below 100 before a sudden drop in 2015. Then, a dramatic outburst occurred from 2018 and broke 300 in 2020. The publication number remained high from 2018 to 2022. As early as 1935, TDs had already been mentioned to be found in the surgical samples from pericolonic and perirectal adipose tissue of colon cancer patients.^4^ The presence of TDs, which was also called metastatic tumour nodules, was found independently related with negative outcome in right‐sided colon cancer patients by Harrison et al. in 1995.^7^ In 1997, Ueno et al. reported “extrabowel skipped cancer infiltration (ex)” as a kind of cancer spread which was similar to TDs of rectal cancer and claimed that ex suggested a worse prognosis.^8^ After that, numerous studies suggested that TDs were closely related to worse outcome and gained TDs more attention in cancer staging, contributing to the sustained academic output in the following years. After the 8th edition of CRC TNM staging from American Joint Committee on Cancer (AJCC) was publish in 2018,^14^ a burst of articles followed, discussing the rationality of the position in this latest guideline.

**TABLE 1:** Main information.

Description	Results
Main information about data	
Timespan	1935:2023
Sources (journals, books, etc)	683
Documents	2147
Annual growth rate %	4.68
Document average age	11.5
Average citations per doc	26.38
References	50,186
Document contents	
Keywords plus (ID)	4384
Author's keywords (DE)	5738
Authors	
Authors	9339
Authors of single‐authored docs	49
Authors collaboration	
Single‐authored docs	64
Co‐authors per doc	6.95
International co‐authorships %	15.46
Document types	
Article	1818
Article, data paper	2
Article, early access	32
Article, proceedings paper	69
Review	226

The average article citation per year had similar trend as annual publication (Figure 2B). The number experienced a period of sudden rise and fall before 1988. Then, the citation number began to rise steadily from 1990 to 2015, followed by a sudden decrease and rose again in 2019.

### 3.2 Analysis of author, country and affiliation

**FIGURE 3:**
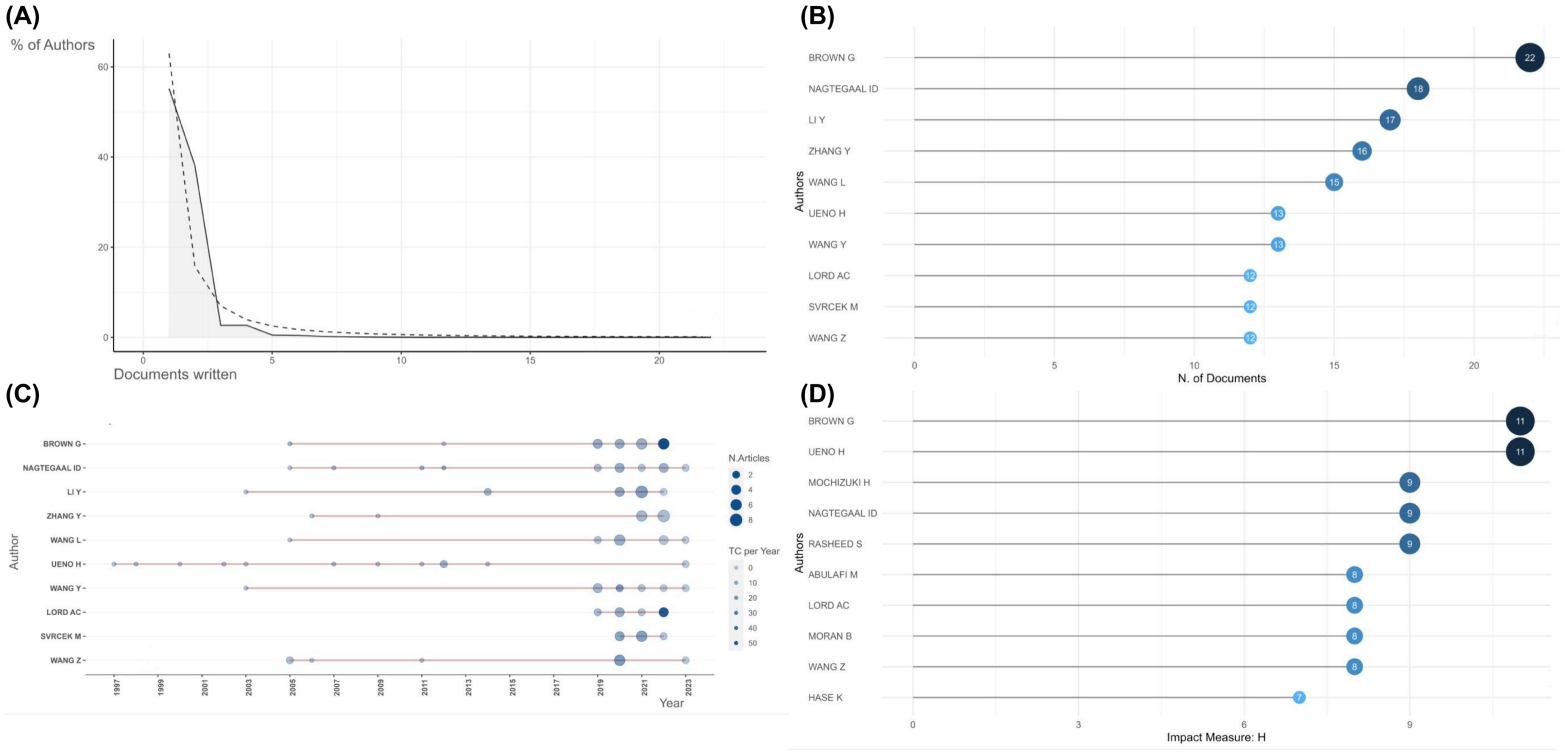
The most impactive authors. (A) Lotka's law. (B) The top 10 most relevant authors. (C) The production of top authors over time. (D) The top 10 most influential author by h‐index.

A total of 9339 authors published articles on TDs in CRC from 1935 to 2023. Studying the authors, which might indicate contributive research groups, could provide meaningful information for researchers. Lotka's law was used to measure the scientific productivity and select the core authors. According to Figure 3A, 55.2% of the authors had published one article and only 1.3% had published at least five articles. Then, the top 10 most relevant authors, based on publication number, are listed in Figure 3B, who at least published 12 articles. Brown G from Royal Marsden Hospital was the most prolific author with 22 publications, followed by Nagtegaal ID, Li Y, Zhang Y, Wang L, with 18, 17, 16, 15 articles, respectively. Figure 3C demonstrates the scientific production of the top 10 most relevant authors over time which could visualize the long‐term contribution of these most prolific authors. The timeline of the researches was shown as the length of the red line. The size and density of the nodes were proportional to the quantity and total citation number per year, respectively. Ueno H stayed the longest in this field and the graphic pattern of Brown G was consistent with the rank based on publication number. Furthermore, the collaboration network between authors is exhibited in Figure S1, in which we found that top authors would collaborate with each other. For example, Brown G, Svrcek M and Rasheed S conducted a Delphi study together to discuss the use of TDs in TNM staging system and direct future precise CRC staging.^29^ Two scholars from Japan, Mochizuki H and Ueno H, reported the clinical significance of cancer nodules in 1997^8^ and contributed to clinical application of TDs in cancer staging collaboratively.^30^

To take both publication quantity and quality into consideration, another ranking based on h‐index was conducted and illustrated in Figure 3D. As illustrated in Figure 3D, Brown G still ranked the first with an h‐index of 11. Ueno H from National Defense Medical College—Japan tied with Brown for the first place. Mochizuki H, Nagtegaal ID and Rasheed S tied for the third whose h‐index was 9. Except for the first place, there seemed to have some fluctuations in ranking comparing to the top 10 relevant author, which might be caused by adding citation numbers into consideration. For example, Ueno H tied with Brown for the first place with an h‐index of 11 and ranked the 6th based on publication number. Nagtegaal ID and Rasheed S both ranked the third with h‐index of 9 and ranked 2 and 8 respectively when it comes to scientific production only.

**FIGURE 4:**
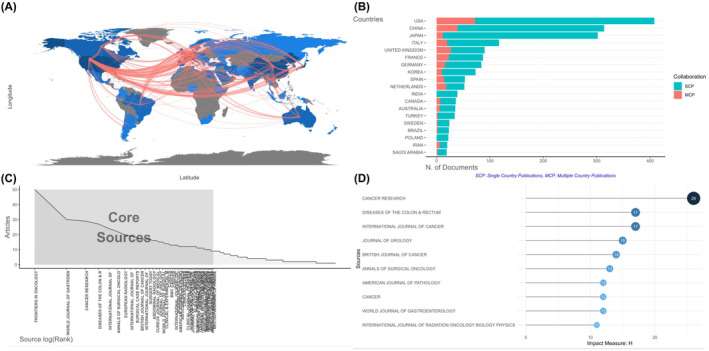
The most influential countries and sources. (A) The collaboration map among countries. (B) The rank of relevant countries based on corresponding author. (C) Bradford's law. (D) The top 10 most influential source by h‐index.

From 1935 to 2023, a total 2147 articles related to TDs in CRC were published worldwide. The exact publication number of each country could be found in Table 2, in which the USA ranked the first with 1445 publications, followed by China, Japan, Italy and France with 1026, 769, 366 and 357 articles published, respectively. A collaboration map among countries is shown in Figure 4A to facilitate the identification of most productive and contributive country. Each red line represented a collaborative relationship between two countries and the width of red lines denoted the collaboration time. Countries which had scientific contribution were marked blue and the number of publications varied according to the depth of blue (number rose with darkness). In Figure 4A, the USA had connections with Europe, China and Japan, among which the relationship between the USA and Europe was the strongest. The various connections suggested that the USA was not only the most prolific but also the most influential country in this field. Co‐author analysis was conducted to further illustrate the proportion of international collaboration. The co‐author's nationality, the number of publications written by authors from the same country, as well as the number of publications written by authors from multiple countries are all displayed in Figure 4B. So, the rate of international collaboration could be indicated by the MCP ratio. As shown in Figure 4B, the USA and China possessed the top two MCP ratio (MCP ratio = 0.176 and 0.124, respectively). European countries also had high MCP ratios, such as Italy, the UK, France, Germany, Spain and Netherlands. The result of co‐author analysis was consistent with the world collaboration map. To further analyse the evolution of research popularity in each country, the country production of top five countries over time is presented in Figure S2A. Generally speaking, the science output of all top five countries were increasing from 1988. Initially, the USA and Japan took the lead in publication number and China exceeded Japan in around 2022, becoming the second largest country of output. Publication might not fully reveal the contribution of these countries to this field since it did not assess the quality of publications. Thus, the top 10 most cited countries are presented in Figure S2B, in which the rank was slightly different from that in Table 2. The USA came in the first in both ranks based on publication number (1445) and citation times (18133), while China came in the third with 1026 articles but 58 citations. The UK ranked the second when it comes to citation number (8324).

**TABLE 2:** Country production.

Region	Freq
USA	1445
China	1026
Japan	769
Italy	366
France	357
UK	347
Germany	267
Netherlands	255
South Korea	237
Canada	158
Spain	149
India	117
Turkey	112
Australia	99
Iran	98
Sweden	76
Brazil	70
Poland	63
Saudi Arabia	56
Romania	55
Mexico	50
Switzerland	46
Egypt	45
Belgium	41
Chile	40
Denmark	38
Ireland	38
Israel	34
Greece	32
Singapore	25
Argentina	24
Tunisia	23
Austria	22
Morocco	18
Pakistan	18
Finland	17
Malaysia	17
Norway	16
Portugal	16
Colombia	13
Hungary	13
Bulgaria	12
New Zealand	12
Croatia	8
Ecuador	8
Jordan	8
Serbia	8
Syria	8
Ethiopia	7
Kuwait	7

Affiliation analysis was carried out for the sake of revealing the cooperation between academic institutions through studying the institutions of the authors. The top 10 most relevant affiliations based on publication number are presented in Figure S2C. Central South University and Memorial Sloan Kettering Cancer Center both came in the first place, each with 46 publications, followed by Yonsei University, SiChuan University and Mashhad University of Medical Sciences with 44, 41, 40 publications, respectively.

### 3.3 Analysis of journal

Bradford's law was adopted to select the core sources and the result is presented in Figure 4C. A total of 48 journals with more than 9 publications on CRC TDs were included in zone 1, which are listed in Table 3. According to Bradford's law, Frontiers in Oncology (IF = 5.738) ranked the first which published 50 articles in this field. Considering both productivity and influence of journals, a rank based on source h‐index is presented in Figure 4D. Cancer Research (IF = 13.312) came into the first place with 26 publications. Diseases of the Colon and Rectum (IF = 4.657) and International Journal of Cancer (IF = 7.316) ranked the second together, each with 17 publications. Difference existed between the rank based on publication only (Table 3) and the rank based on h‐index (Figure 4D), especially for the top two journals. Thus, the over‐time dynamic of publication of top five journals based on productivity was studied and the result is shown in Figure S2D. Cancer Research and Diseases of the Colon and Rectum were the first two journals began to publish articles on colorectal TDs. Later, around 2000, the publications in World Journal of Gastroenterology (IF = 5.374) began to rise. Around 2019, the publication number of Frontiers in Oncology and International Journal of Surgery Case Report increased dramatically, quickly exceeding the other three journals.

**TABLE 3:** Bradford's law.

Journal	Rank	Freq	CumFreq	Zone
Frontiers in Oncology	1	50	50	Zone 1
World Journal of Gastroenterology	2	30	80	Zone 1
Cancer Research	3	29	109	Zone 1
Diseases of the Colon & Rectum	4	27	136	Zone 1
International Journal of Surgery Case Reports	5	24	160	Zone 1
Annals of Surgical Oncology	6	22	182	Zone 1
European Radiology	7	20	202	Zone 1
International Journal of Cancer	8	19	221	Zone 1
Surgical Case Reports	9	18	239	Zone 1
British Journal of Cancer	10	17	256	Zone 1
International Journal of Colorectal Disease	11	17	273	Zone 1
Surgery Today	12	17	290	Zone 1
Abdominal Radiology	13	16	306	Zone 1
Cureus Journal of Medical Science	14	16	322	Zone 1
Journal of Urology	15	15	337	Zone 1
World Journal of Surgical Oncology	16	15	352	Zone 1
Clinical & Experimental Metastasis	17	14	366	Zone 1
Cureus	18	14	380	Zone 1
BMC Cancer	19	13	393	Zone 1
Cancer	20	13	406	Zone 1
International Journal of Radiation Oncology Biology Physics	21	13	419	Zone 1
Modern Pathology	22	13	432	Zone 1
American Journal of Pathology	23	12	444	Zone 1
Anticancer Research	24	12	456	Zone 1
Cancer Letters	25	12	468	Zone 1
Cancers	26	12	480	Zone 1
Clinical Journal of Gastroenterology	27	12	492	Zone 1
Colorectal Disease	28	12	504	Zone 1
International Journal of Molecular Sciences	29	12	516	Zone 1
Journal of Surgical Oncology	30	12	528	Zone 1
Medicine	31	12	540	Zone 1
Scientific Reports	32	12	552	Zone 1
Surgical Endoscopy and Other Interventional Techniques	33	12	564	Zone 1
American Journal of Surgical Pathology	34	11	575	Zone 1
Histopathology	35	11	586	Zone 1
Japanese Journal of Cancer Research	36	11	597	Zone 1
Journal of Nuclear Medicine	37	11	608	Zone 1
Oncogene	38	11	619	Zone 1
World Journal of Surgery	39	11	630	Zone 1
American Journal of Case Reports	40	10	640	Zone 1
British Journal of Surgery	41	10	650	Zone 1
Hepato‐Gastroenterology	42	10	660	Zone 1
Journal of Clinical Oncology	43	10	670	Zone 1
PLoS One	44	10	680	Zone 1
World Journal of Clinical Cases	45	10	690	Zone 1
American Journal of Roentgenology	46	9	699	Zone 1
Annals of Surgery	47	9	708	Zone 1
British Journal of Radiology	48	9	717	Zone 1
Diagnostic Cytopathology	49	9	726	Zone 2
EJSO	50	9	735	Zone 2

### 3.4 Analysis of document and reference

**FIGURE 5:**
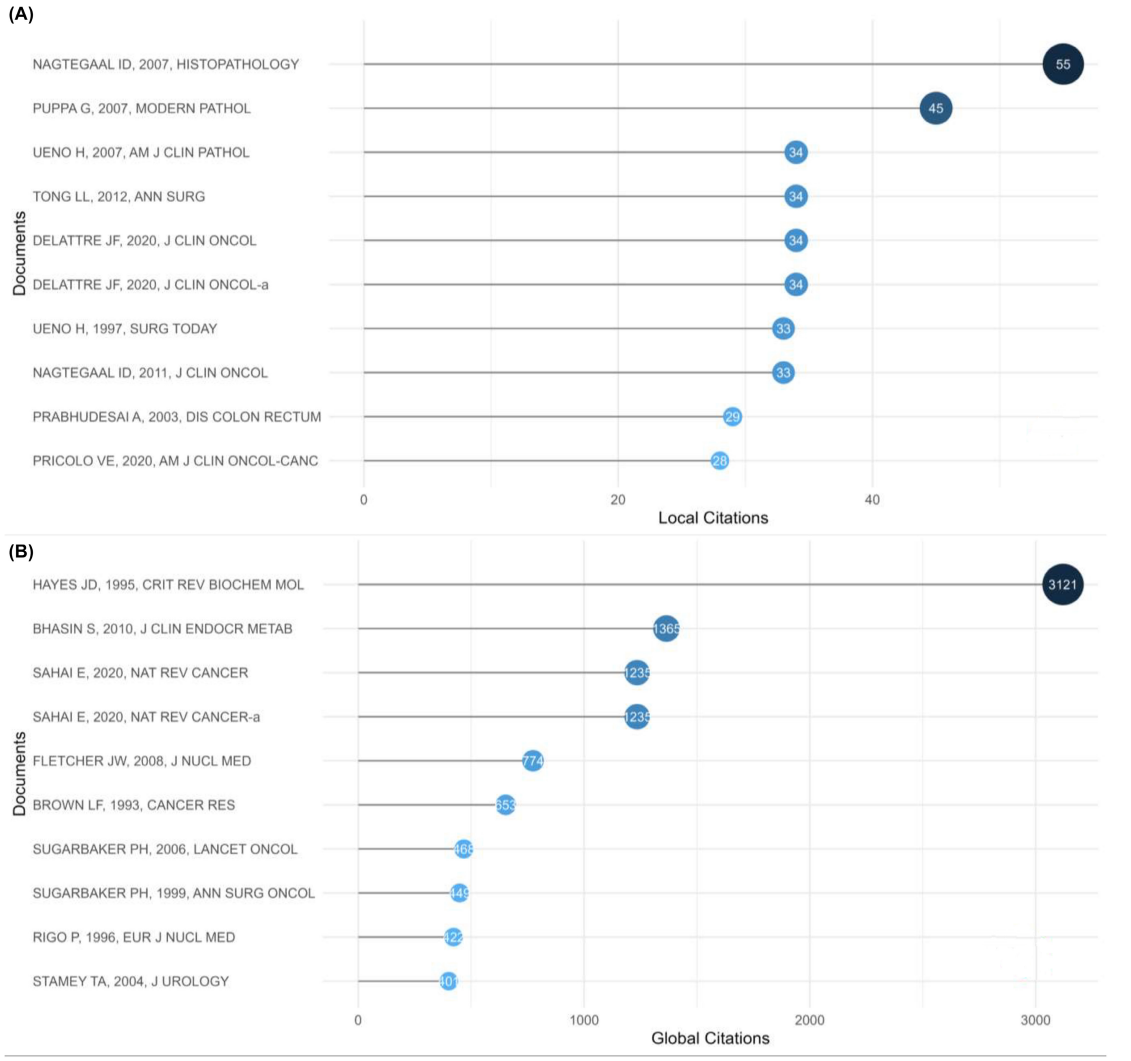
The document analysis. (A) The top 10 most local cited documents. (B) The top 10 most global cited documents.

It was asserted that a work's citation rate might indicate both its influence or importance as well as its acceptance within the field of science.^31^ As a result, the highly cited papers were identified as a hint of the hot spot in this field from 1935 until now. Local citations show the impact of a specific article in our retrieval collection, whereas global citations show the influence of an article across the entire database. Both local and global cited documents was listed (Figure 5A,B). To avoid the drift of the research focus brought by articles outside the retrieved collection, the top 10 most local cited documents were focused on. Colorectal tumour deposits in the mesorectum and pericolon; a critical review (Nagtegaal et al., 2007) and Pathological assessment of pericolonic TDs in advanced colonic carcinoma: relevance to prognosis and tumour staging (Puppa et al., 2007) ranked the top two most locally cited with 55 and 45 times of citation (Figure 5A). Also, these two articles were the 5th and 6th most locally cited references (Figure S3A) indicating their importance as cornerstones in this field.

### 3.5 Analysis of keywords network

**FIGURE 6:**
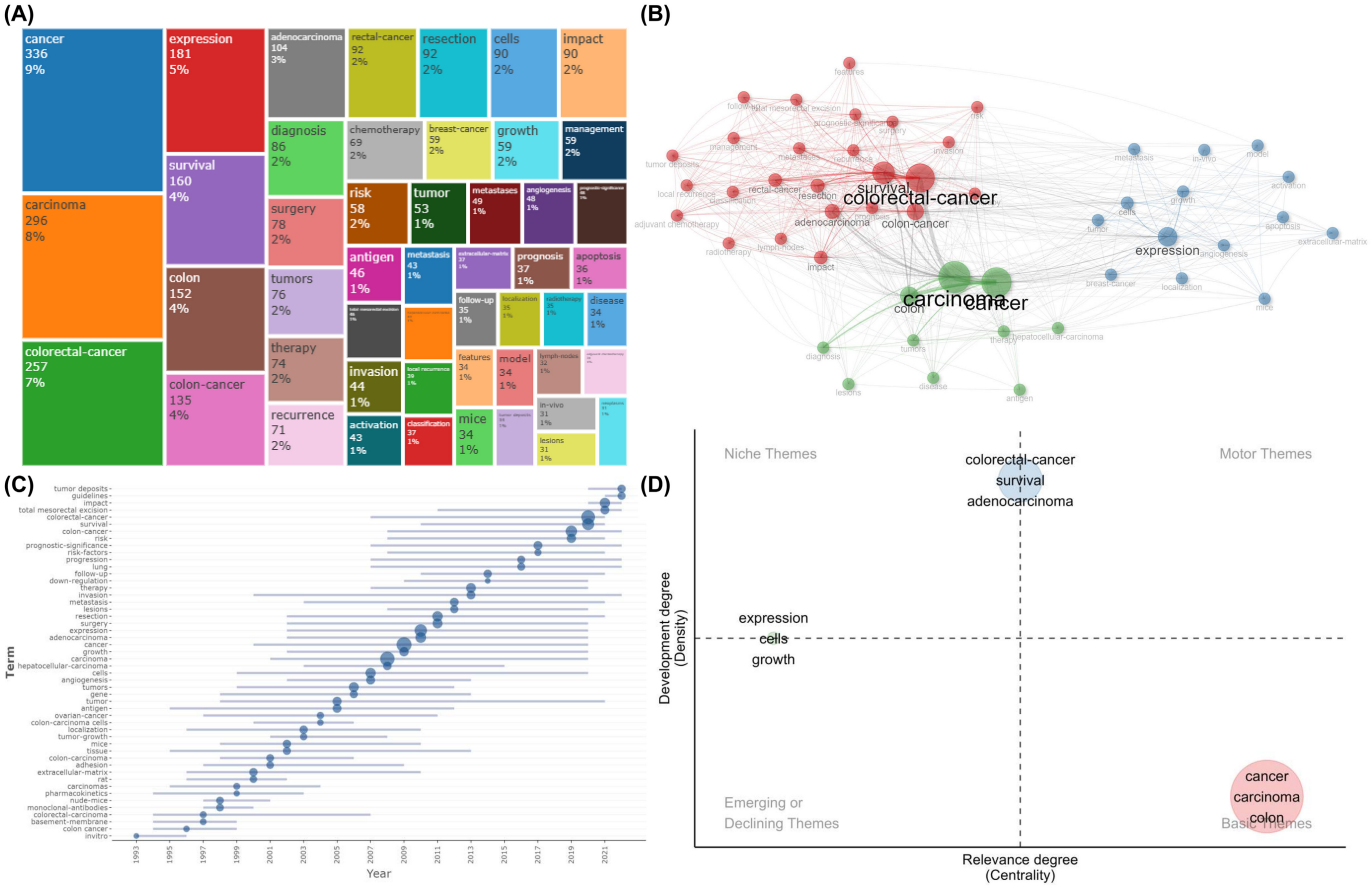
The keyword and trend analysis. (A) The tree map of the top 50 most frequently occurred keywords. (B) The keyword co‐occurrence analysis. (C) The trend topics map. (D) The thematic map.

It was a common knowledge that well‐crafted keywords could concisely describe the article topics and provide readers with a thorough summary of the literature. Therefore, certain main challenges in a discipline could be demonstrated and research hotspots in the specified field could be found using high‐frequency keywords retrieved from current publications using bibliometric approaches. A total of 5738 authors' keywords were extracted from 2147 documents (Table 1). The top 50 most frequently occurred keywords were listed on a tree map. Word “cancer”, “carcinoma”, “colorectal cancer” were the top three most frequently occurring words with 336, 296, 257 occurrences, respectively. “Expression” and “survival” came in the fourth and the fifth place with 181 and 160 occurrences. “Colon”, “colon cancer” and “adenocarcinoma” ranked from the sixth to the eighth. The word “rectal cancer” tied with “resection” for the ninth place (Figure 6A).

Figure S3B presents the key word dynamic for the sake of evaluating the evolution of the research hot topics from 1990 to 2023. Generally, the top 10 keywords all kept growing since 1990. “Cancer” and “carcinoma” kept taking the lead as general subjects of this fields and reached 332 and 292 times in 2023. Word “expression” maintained steady growth since 1990 and was exceeded by “colorectal cancer” in 2020. “Rectal cancer”, “colon” and “colon cancer” also experienced slight increase in growing speed in 2019, indicating that the focus in intestinal cancer was further refined.

A Sankey diagram was drawn for further analysis on varied levels of attention received by the keywords in different periods of time for the sake of identifying different research hotspots each time period (Figure S3C). Before 2000, the most frequent keywords included “in vivo”, “extracellular‐matrix”, “tissue”, “human‐colon”, “cancer”, “positron‐emission‐tomography”, “complications”. After 2000, “expression” and “classification” appeared. Since 2011, treatment related keywords “radiation‐therapy” and “endoscopic submucosal dissection” enriched the content.

The degree of interconnection among keywords as well as their internal relationships were explained by keywords co‐occurrence analysis, which might aid researchers in understanding the frontiers and the evolution of this field. The keyword co‐occurrence map was built on keywords that appeared in the same article together. A clustering process was conducted to group closely connected keywords and the identified group might indicate the core research focus. A total of 49 words were included in keyword co‐occurrence analysis after quality control. The connection, closeness and frequency of keywords were visualized in co‐occurrence map (Figure 6B). In Figure 6B, each node represented a keyword. The nodes' sizes reflected the quantity of publications with relating keywords in this field, while the nodes' colours indicated the corresponding clusters in the map. In Figure 6B, three clusters were identified and were coloured in red, blue and green, respectively, indicating that there were three research themes in colorectal TDs. The red cluster was the biggest, including adenocarcinoma, adjuvant chemotherapy, chemotherapy, classification, colon cancer, colorectal cancer, features, follow‐up, impact, invasion, local recurrence, lymph nodes, management, metastases, prognosis, prognostic significance, radiotherapy, rectal cancer, recurrence, resection, risk, surgery, survival, total mesorectal excision and TDs. The blue cluster consisted of activation, angiogenesis, apoptosis, breast‐cancer, cells, expression, extracellular‐matrix, growth, in vivo, localization, metastasis, mice, model and tumour. The smallest cluster was coloured in green, which was made up of antigen, cancer, carcinoma, colon, diagnosis, disease, hepatocellular‐carcinoma, lesions, therapy and tumours. The connection between two words was represented by the line, so it was clear that words such as “cancer”, “colorectal cancer”, “survival” and “expression” had strong correlation with other words within or out of its cluster.

### 3.6 Analysis of trend topics and themes

Generally speaking, topics and themes could indicate the research hotspots in a certain time period. As a result, trend topics and themes could provide us with the information about the evolution of a certain field over time. Topics with a minimum frequency of nine were incorporated into analysis and visualized in the trend topics map (Figure 6C). A total of 50 topics were selected, ranging from 1993 to 2022, and the detailed frequency and time range could be found in Table S1. “TDs”, “impact”, “guideline” all emerged around 2020 and appeared 34, 90, 29 times, respectively, indicating that the clinical application of TDs integrated in clinical guidelines has been gaining more and more attention in recent years and may remain a promising topic in the future. Furthermore, “total mesorectal excision”, “survival”, “prognostic significance”, “risk factors”, “progression”, emerged as a hot topic much earlier, around 2007–2010, and are still occurring at high frequencies, suggesting the researching for indicators for CRC's prognosis and treatment options have long been a research focus and may still be a popular research direction.

The thematic map in Figure 6D was drawn to further reveal the current developing status of the research themes. Its X‐axis showed centrality and Y‐axis represented density, reflecting the degree of the theme's significance and the degree of development, respectively. The map was divided into four equal parts. At the top left located niche themes that were highly developed and isolated with good development but less important for the field now. Themes in the top right were both significant and well‐developed. The situation in the bottom left might be a little bit complicated since the themes there were either emerging or declining, meaning that they were less developed and had little significance. Basic themes, which were of great importance to the subject but were still less developed, were placed in the bottom right quadrant, which referred to fundamental concepts. The red cluster of themes contained “cancer”, “carcinoma”, “colon” was of great importance and still needed further study, indicating that the detailed criteria for careful diagnosis was not yet complete. The blue cluster consisted of “survival”, “colorectal cancer” and “adenocarcinoma” were of medium importance and had already been extensively studied, meaning that the prognostic value of TDs were well established and reached a consensus in this field. “Expression,” “cells,” and “growth” were coloured in green and were placed in a less important status.

## 4 DISCUSSION

CRC remained a prevalent mailgnancy with significant global incidence and mortality rates.^1^ The tradition TNM staging system plays a crucial role in its treatment decision and prognosis prediction.^3^ First of all, our concise review elucidated the hisorical evolution of TDs' utilization in cancer staging system. In the 5th edition of the AJCC Cancer Staging Manual, isolated TDs with a diameter >3 mm were classified into N category and the rest belonged to T categories^11^; however, subsequent studies revealed significant survival impact irrespective of size, challenging the validity of the 5th edition.^32^ Subsequently, the size determined criteria were replaced by contour‐based criteria in the 6th edition, where tumour nodules with smooth and regular contours were regarded as lymph nodes metastases and integrated in N category, while nodules irregular contours were classified in T category as a type of vascular invasion.^12^ But the ability of this version to distinguish the origin of TDs and the rationality of integrating TDs in T and N category instead of M category were questioned.^33^ Satellite tumour nodules without remaining lymph nodes were categorized in the 7th edition as discontinuous spread or venous invasion with extravascular spread, falling under the “N1c” category. N1c could advance the staging result to stage III, even in the absence of nodal metastases. The quantity of TDs could be mentioned as site‐specific factors without changing the N stage.^5^, ^13^ However, the 7th edition still entrusted the definition of TDs to pathologists' own judgement without clear guidelines, which might lead to staging drifts and subsequent treatment variations.^15^ Ignoring the number of TDs could overlook valuable hidden information.^6^ In the latest 8th edition, TDs refers to tumour foci absent of discernible residual lymph node/vascular/neural structures tissue, located in the pericolic or perirectal fat, away from the tumour invasion front, in the lymphatic drainage area of the tumour.^3^, ^14^ The number of TDs was still recorded in site‐specific factors, together with the origin of TDs, designated as L+ for lymphatic/small‐vein invasion; V+ for deposits with accompanying smooth muscle cells or red blood cells in endothelial cell‐lined areas, and perineural invasion for deposits around neural structures. TDs were still classified in N1c category based on their sites.^14^ However, unresolved issues persist, including TDs interobserver disagreement and queries regarding excluding intravascular or perineural TDs, which showed no increase in prognostic value. The N1c category was still questioned for ignoring of the quantity of TDs and new staging strategy counting TDs similarly to LN gaining attention.^16^ In brief, a more objective assessment criteria of TDs was needed. The origin of TDs, bearing valuable information, remains difficult to define. Furthermore, a revised cancer staging system is necessary, wherein the number and origin of TDs can achieve the full prognostic value. Then, we conducted a systemic bibliometric analysis coupled with a review to further elucidate the research focus and progress in this field.

### 4.1 General information

A total of 2147 papers on TDs in CRC from 1 January 1935 to 30 April 2023 were collected from WOS for bibliometric analysis. Professor Gina Brown emerged as the most prolific and influential author in this field. As a radiologist and professor in gastrointestinal cancer imaging at the Royal Marsden and Imperial College London, Professor Brown has been devoted to radiological tests of TDs and their application in cancer staging. In 2005, Brown published an article proposing a high spatial resolution imaging technique to access the extramural infiltration depth, TDs, and the possibility of positive circumferential margin involvement before surgery.^34^ After the 8th edition of TNM came out, Brown's group conducted a meta‐analysis criticizing TNM 8 for failing to correct the underestimation of adverse prognosis in patients due to setting TDs in an inferior status compared to lymphatic nodes metastasis. The meta‐analysis also pointed out the lack of solid evidence in the exclusion criteria for TDs. Furthermore, it is reported that in the presence of extramural venous invasion (EMVI), the probabilities of developing extranodal tumour deposits (ENTDs) were demonstrably higher, which might indicate metastases pathway.^13^ Recently, Brown's group innovatively classified magnetic resonance lymph node metastases (mrLNMs) and magnetic resonance TDs (mrTDs) and linked them to outcome independently in rectal cancer. Previous assessments combined them as a single concept of “nodes,” potentially leading to lose of valuable prognostic information. The study implied that LNMs failed to predict either pathological nodal status or poor survival after being distinguished from TDs on MRI. The predictive accuracy of positive mrTD/mrEMVI status was higher, suggesting its advantage in selecting proper treatment and follow‐up regimens.^35^

The United States was identified as the most productive and influential country in the field of colorectal TDs. While China was prolific, it was less influential. Central South University from China and Memorial Sloan Kettering Cancer Center from America both ranked first as the most prolific affiliation. Frontiers in Oncology (IF = 5.738) was the most productive source, while Cancer Research (IF = 13.312) ranked first based on contribution.

Document analysis helped locate the most influential documents and get a quick overview of the landmarks in the field. Ten documents were cited greater than or equal to 28 times locally, and ten documents were cited more than 400 times globally, indicating significant attention to colorectal TDs and prosperous development in this area. The first most cited document was “Colorectal tumour deposits in the mesorectum and pericolon; a critical review” by Nagtegaal, followed by “Pathological assessment of pericolonic TDs in advanced colonic carcinoma: relevance to prognosis and tumour staging” from Puppa.^33^, ^36^

### 4.2 Research hotspots and trend topics

The result of keywords co‐occurrence analysis demonstrated three clusters.

#### 4.2.1 Cluster 1: the application of TD in TNM staging system

Coloured in red was the largest cluster identified by co‐word analysis, including “survival,” “colorectal cancer,” “TDs,” “recurrence,” “prognostic significance,” “adjuvant chemotherapy,” “surgery,” “management”. This indicated that the clinical significance and prognostic value of TDs have been a significant research hotspot, contributing to tumour staging and guiding treatment selection, such as surgical resection or adjuvant therapy. Recent trend topics, including “impact”, “guideline”, “total mesorectal excision”, “survival”, “prognostic significance”, “risk factors”, “progression”, are all related to this dominant research hotspot.

Since the discovery of TDs in 1935, their pathological classification and application in the TNM staging system have garnered significant attention and scientific output. Numerous clinical studies have verified the negative prognostic value of TDs. For instance, in 1995, Harrison et al. substantiated that metastatic tumour nodules were independent prognostic indicator negatively related to the outcome of right‐sided colon cancer patients.^7^ Similarly, in 1997, Hideki Ueno and Hidetaka Mochizuki reviewed 369 rectal adenocarcinoma patients and found that TDs were significantly associated with worse outcome, suggesting their potential negative prognostic value.^8^ In 1997, the integration of TDs into the TNM staging system has evovled over time. In the 5th edition (TNM5), TDs with a diameter larger than 3 mm were identified as LNMs and categorized into the N category.^13^ However, the classification rule was later questioned for lacking substantiating clinical data. Subsequent editions, such as TNM6, focused on the morphological characteristics of TDs instead of size, but the criteria were debated for their lack of substantiating clinical data and poor reproducibility.^36^ New categorization criteria propsosed by Ueno et al. Aimed at improving TNM staging system, in which extranodal cancer deposits were further subdivided into vascular invasion‐type^37^ and non‐vascular invasion‐type. The highest prognostic accuracy was achieved when non‐vascular invasion‐type deposits were integrated into the N category and vascular invasion‐tyoedeposits were categorized as T factor.^30^ Later in 2010, the 7th edition of TNM staging system (TNM7) integrated the quantity of TDs into site‐specific prognostic markers and the presence of TDs was classified as N1c category in T1‐2 lesions with negative LNM. TDs, regardless of their various origins, were integrated into N category in the absence of LNM. TNM7 further affirmed the association between the presence of TDs and worse outcome reported in previous studies.^33^, ^36^, ^38^ However, unresolved issues persist. In the latest TNM staging system (TNM8) introduced in 2008, TDs were considered based on their origin and quantity but remained integrated into site‐specific factors rather than the staging system itself. Despite this update, TNM8 left several problems unresolved. First of all, patients with both positive TDs and LNM exhibited worse prognosis, yet TDs were disregarded when LNM was present, raising concerns about missing prognostic information. Additionally, the quantity of TDs was overlooked in stage determination, despite evidence suggesting their independent prognostic value. Number of TDs had long been considered as a prognostic factor. However, TNM8 overlooks this aspect in staging determination, despite evidence suggesting that TDs ≥3 could serve as an indicator for worse prognosis, independent of LNM status.^39^ Incorporating TDs quantity into staging criteria, as advocated by recent studies, would enhance prognostic accuracy.^10^

Considering the clinical prognostic value of TDs, the place of TDs in TNM staging system was modified for several times, despite still being suboptimal. The review summarized current unresolved problems in the application of TDs in TNM. First, the definition of TDs remains controversial, with different criteria proposed across editions. Second, the staging strategy of TDs is still not optimal, as they are considered inferior to LNM despite their strong prognostic value.^39^, ^40^, ^41^ Third, under current staging criteria, a systematic lymphadenectomy might be necessary to fully evaluate TDs post‐operation and the appilcation of TDs in TNM staging heavily relies on pathological studies.^42^ Finally, the prognostic value of TDs in the context of neoadjuvant therapy remains to be fully elucidated, as their nature in post‐neoadjuvant patients is still under debate^41^, ^43^, ^44^ In conclusion, despite modifications to the TNM staging system, challenges persist in the application of TDs. Future research should focus on refining the defining the definition and staging strategy of TDs, exploring methods for preoperative analysis, and investigating their prognostic value in the context of evolving treatment modalities.

#### 4.2.2 Cluster 2: the pathogenesis of TDs

Coloured in blue was a smaller cluster of topics focusing on the mechanism of TDs, including cellular processes, gene expression, growth patterns, in vivo, localization, metastasis and animal model etc. Despite its existence, this cluster has seen limited progress, as evidenced by thematic maping and word trends. Studies in the pathogenesis of TDs were scarce and the cause of TDs was still a mystery waiting to be uncovered.^45^ Several researches which reported certain changes at genetic level related to TDs. Dai et al. conducted an immunohistochemical analysis to discover the relationship between the expression levels of eight EMT related proteins and clinicopathologic characteristics, including TDs. The result showed that lower expression of E‐cadherin and claudin‐1, along with elevated levels of CA19–9 in serum, correlated with the presence of TDs.^46^ Similarly, another immunohistochemical study including 345 CRC patients revealed the correlation between loss of Yin Yang 1 (YY1) expression and TDs presence.^47^ Furthermore, Zhang et al. conducted a retrospective study on the relationship between KRAS status and TDs in 45,761 CRC patients, suggesting an independent association between KRAS and TDs presence, with an odds ratio of 1.11 (95% CI 1.02–1.20).^48^ More recently, Xiao et al. constructed a nomogram consisted of six relative microRNAs purported to predict TDs status. Among these microRNAs, miR‐614, miR‐1197 and miR‐4770 were up regulated, while miR‐3136, miR‐3173, and miR‐4636 were downregulated.^45^ Additionally, Liu et al. observed lower expression levels of SATB2 in the presence of TDs.^49^ These studies shed light on potential molecular mechanisms underlying TDs formation, yet further research is warranted to elucidate the complex pathogenesis of TDs in CRC.

#### 4.2.3 Cluster 3: the assessment of TDs

Coloured in green was the smallest cluster of topics including words such as “diagnosis”, “antigen”. Despite its size, this cluster is crucial as it delves into the preoperative analysis and diagnosis of TDs, a domain still shrouded in mystery. In 1992, Davidson used radiation‐labelled monoclonal antibodies targeting epithelial membrane antigen (EMA) for preoperative analysis of CRC. However, the findings regarding metastatic deposits were less than satisfactory.^50^ Similarly, Brown G, an expert in preoperative imaging of TDs in CRC patient, proposed a technique in 2005 to attain high special resolution for TDs analysis before surgery. The article described essential factors to consider when taking and analysing MRI images.^34^ Furthermore, Lord et al. Utilized MRI to assess TDs and EMVI status, asserting that these statuses could provide valuable insights for treatment and follow‐up planning. The author advocated for the use of chemoradiotherapy in TDs/EMVI positive rectal cancer patients.^35^ For the sake of precise treatment, the assessment of TDs before treatment holds significant value. Thus, preoperative techniques for TDs analysis, such as MRI imaging, might be a hotspot in the future.

Our study analysed the research landscape of TDs in CRC. We summarized the current development of this research field, especially the prognostic value and the application of TDs in TNM staging system. We also pointed out the research vacancies in the pathogenesis and the preoperative assessment of TDs, which might act as a guidance for future research. However, the fault and deviation were inevitable due to the data retrieving mechanism. Also, only the relevant articles came from WOS Core Collection database were included in our study which might lead to incomplete acquisition of pertinent articles. To acquire data more thoroughly for future research, we might merge the documents from several other databases, such as PubMed, Embase. In addition, since we were unable to include relevant articles published after the data retrieving, the timeliness might be influenced. Furthermore, we should seek guidance from essential scholars in professional knowledge involved in the literature we obtained.

## 5 CONCLUSION

**FIGURE 7:**
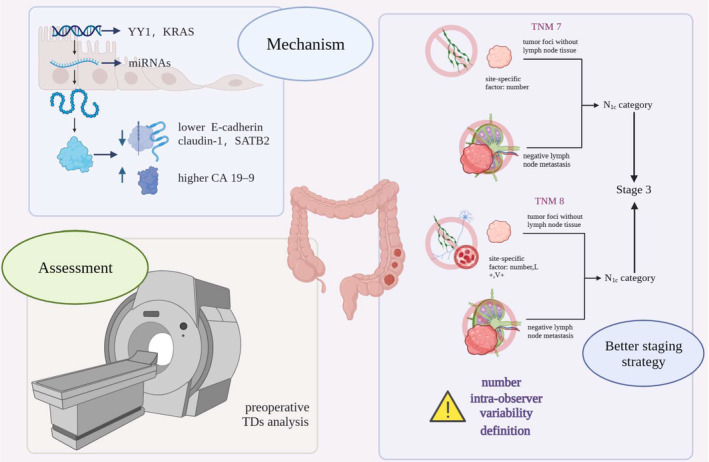
Mechanism diagram of the application of TDs in TNM staging system.

All in all, our study demonstrated the evolution of the application of TDs in TNM staging system with some unsolved problems and emerging hotspots, as was summarized in a mechanism diagram (Figure 7). First, the definition of TDs still needed a firm guideline, instead of solely depending on the experience and judgement of pathologists. Second, the TNM system still need to be refined, for the sake of a more accurate prognosis and a wiser treatment strategy. The status of TDs in TNM was still inferior to LNM, despite that TDs showed strong prognostic value in previous researches. The number of TDs and the origin of TDs remain to be better integrated into TNM. Third, current TDs evaluation is still preoperative, which mostly depend on need a systemic lymphadenectomy during the surgery. Thus, the noninvasive preoperative evaluation of TDs by MRI might be a future hotspot, which may guide the choice of a more precise treatment strategy. Also, due to the rise of the neoadjuvant therapy, the interpretation of TDs after neoadjuvant therapy still needs to be clarified. Lastly, the field of pathogenesis of TDs is still nearly empty. The molecular mechanism of TDs remains to be clarified to identify the source of TDs, guide future precision therapy, and further improve the accuracy of prognosis. Nonetheless, more studies are still needed in future. The most influential authors, affiliations and journals can help academics to find cutting‐edge research of the highest quality. The most cited documents help to grasp the core of this evolving field. In brief, our study summarized the development of this field and guided future research directions.

## AUTHOR CONTRIBUTIONS

**Zhengyan Chang:** Data curation (equal); formal analysis (equal); funding acquisition (equal); writing – original draft (equal); writing – review and editing (equal). **Huijun Fu:** Data curation (equal); formal analysis (equal); writing – original draft (equal); writing – review and editing (equal). **Jiaqi Song:** Data curation (equal); formal analysis (equal); writing – original draft (equal); writing – review and editing (equal). **Cheng Kong:** Data curation (equal); formal analysis (equal); writing – original draft (equal); writing – review and editing (equal). **Ruting Xie:** Data curation (equal); formal analysis (equal); writing – original draft (equal); writing – review and editing (equal). **Man Pi:** Data curation (equal); formal analysis (equal); writing – original draft (equal); writing – review and editing (equal). **Xuechen Sun:** Data curation (equal); formal analysis (equal); writing – original draft (equal); writing – review and editing (equal). **Wentao Zhang:** Data curation (equal); formal analysis (equal); writing – original draft (equal); writing – review and editing (equal). **Yifan Liu:** Data curation (equal); formal analysis (equal); writing – original draft (equal); writing – review and editing (equal). **Ruizhi Huang:** Data curation (equal); formal analysis (equal); writing – original draft (equal); writing – review and editing (equal). **Tingsong Yang:** Data curation (equal); formal analysis (equal); writing – original draft (equal); writing – review and editing (equal). **Dongyan Han:** Data curation (equal); formal analysis (equal); writing – original draft (equal); writing – review and editing (equal).

## FUNDING INFORMATION

This study was supported in part by the National Natural Science Foundation of China (No. 82002923); Shanghai Rising‐Star Program (Sailing Special Program) (No. 23YF1458400).

## CONFLICT OF INTEREST STATEMENT

The authors declare that the research was conducted in the absence of any commercial or financial relationships that could be construed as a potential conflict of interest.

## Supporting information


Figure S1.
Figure S2.
Figure S3.

Table S1.

## Data Availability

Not applicable.
