# Supplementary material for: The past, present, and future of tumour deposits in colorectal cancer: Advancing staging for improved prognosis and treatment decision‐making
Source: J Cell Mol Med. 2024 Aug 27;28(16):e18562. doi: 10.1111/jcmm.18562 (PMC11348060; doi:10.1111/jcmm.18562)
Supplement: Supplementary file 1 — Figure S1. Figure S2. Figure S3. [file JCMM-28-e18562-s002.docx]

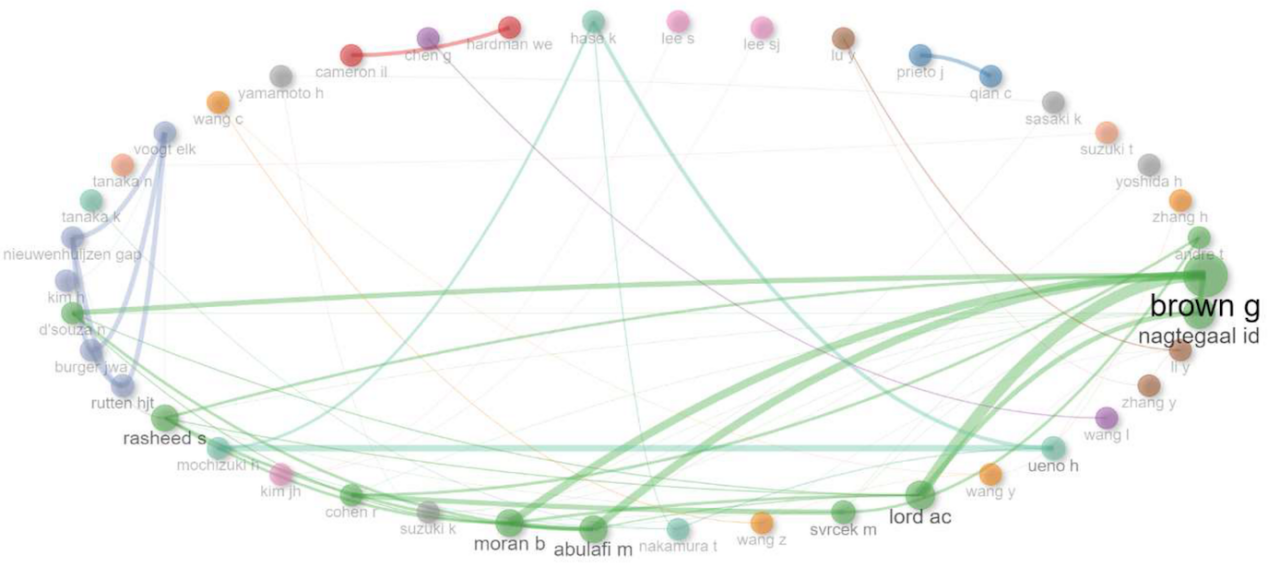


## Figure S1 The collaboration map between authors.


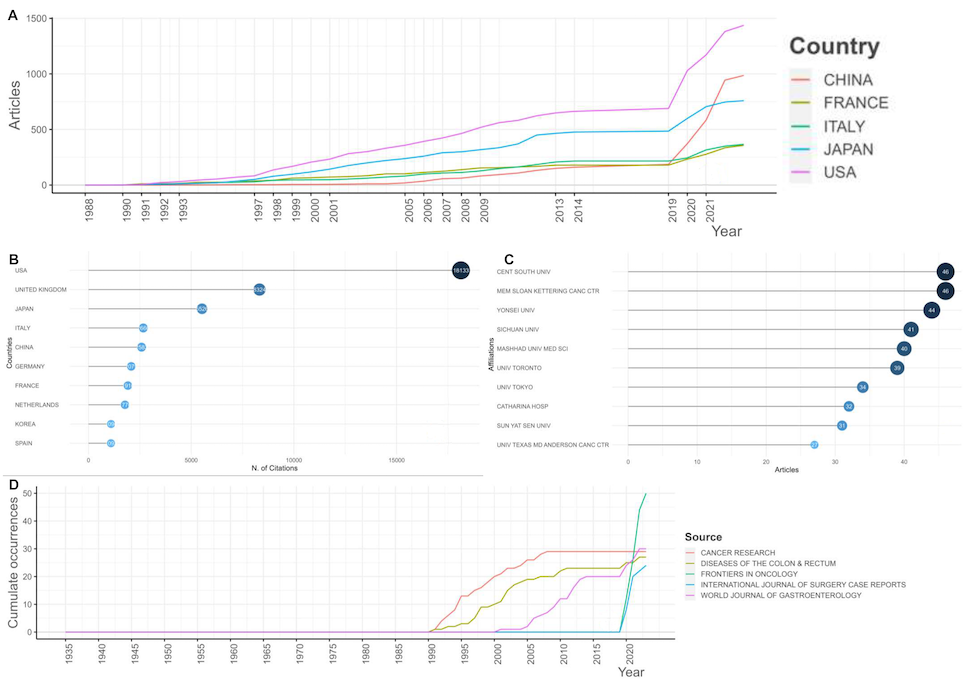


## Figure S2 The supplementary bibliometric indicators analysis.

## (A)The production of top 5 productive countries over time. (B)The top 10 most cited countries. (C)The top 10 most relevant affiliations. (D)The growth of top 5 journals over time.


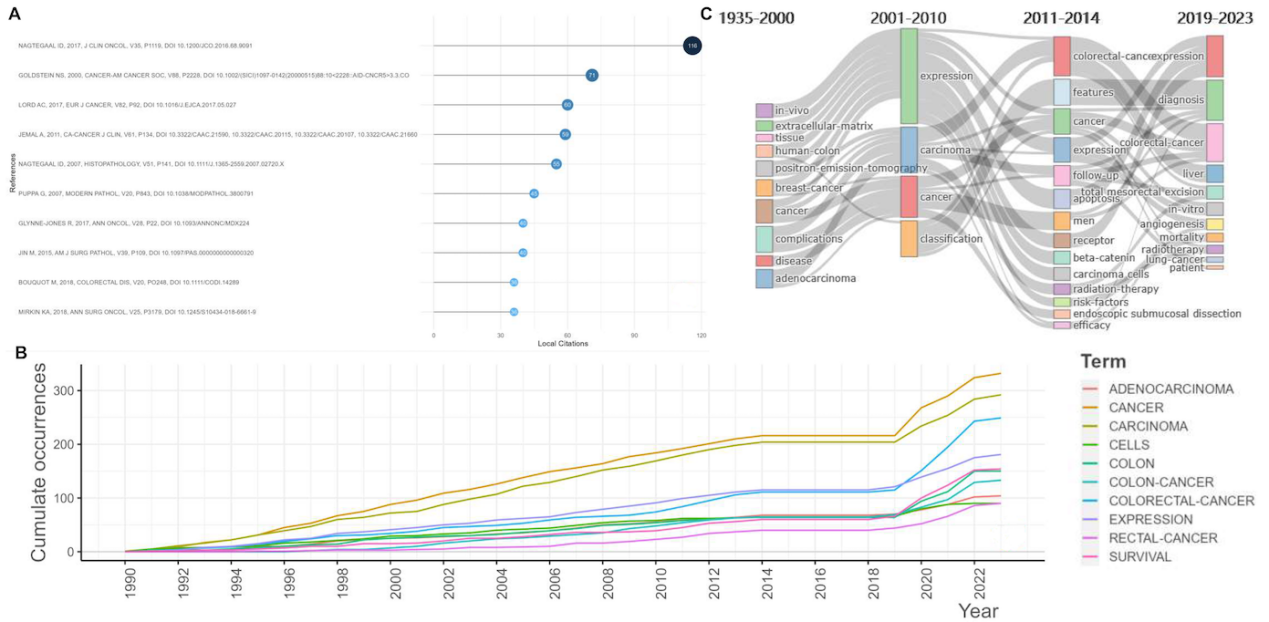


## Figure S3 The supplementary document and keyword analysis.

## (A)The top 10 most local cited references. (B)The key word dynamic. (C)Sankey diagram for keyword evolution.
