# Supplementary material for: The past, present, and future of tumour deposits in colorectal cancer: Advancing staging for improved prognosis and treatment decision‐making
Source: J Cell Mol Med. 2024 Aug 27;28(16):e18562. doi: 10.1111/jcmm.18562 (PMC11348060; doi:10.1111/jcmm.18562)
Supplement: Supplementary file 2 — Table S1. [file JCMM-28-e18562-s001.docx]

**Supplementary Table 1. The trend topics**

| **item** | **freq** | **year_q1** | **year_med** | **year_q3** |
| --- | --- | --- | --- | --- |
| invitro | 9 | 1993 | 1993 | 1996 |
| colon cancer | 11 | 1994 | 1996 | 1999 |
| colorectal-carcinoma | 15 | 1994 | 1997 | 2007 |
| basement-membrane | 13 | 1994 | 1997 | 1999 |
| monoclonal-antibodies | 24 | 1997 | 1998 | 2000 |
| nude-mice | 24 | 1997 | 1998 | 2001 |
| carcinomas | 15 | 1995 | 1999 | 2004 |
| pharmacokinetics | 10 | 1994 | 1999 | 2003 |
| extracellular-matrix | 37 | 1996 | 2000 | 2010 |
| rat | 20 | 1996 | 2000 | 2002 |
| colon-carcinoma | 25 | 1998 | 2001 | 2006 |
| adhesion | 22 | 1997 | 2001 | 2009 |
| mice | 34 | 1998 | 2002 | 2010 |
| tissue | 29 | 1995 | 2002 | 2013 |
| localization | 35 | 1996 | 2003 | 2010 |
| tumor-growth | 18 | 2001 | 2003 | 2008 |
| ovarian-cancer | 18 | 1997 | 2004 | 2011 |
| colon-carcinoma cells | 11 | 2000 | 2004 | 2006 |
| tumor | 53 | 1998 | 2005 | 2021 |
| antigen | 46 | 1995 | 2005 | 2012 |
| tumors | 76 | 1999 | 2006 | 2012 |
| gene | 29 | 1998 | 2006 | 2013 |
| cells | 90 | 1999 | 2007 | 2020 |
| angiogenesis | 48 | 2002 | 2007 | 2013 |
| carcinoma | 296 | 2001 | 2008 | 2020 |
| hepatocellular-carcinoma | 40 | 2003 | 2008 | 2015 |
| cancer | 336 | 2000 | 2009 | 2020 |
| growth | 59 | 2002 | 2009 | 2020 |
| expression | 181 | 2002 | 2010 | 2020 |
| adenocarcinoma | 104 | 2002 | 2010 | 2020 |
| resection | 92 | 2002 | 2011 | 2021 |
| surgery | 78 | 2002 | 2011 | 2020 |
| metastasis | 43 | 2003 | 2012 | 2021 |
| lesions | 31 | 2008 | 2012 | 2020 |
| therapy | 74 | 2007 | 2013 | 2020 |
| invasion | 44 | 2000 | 2013 | 2022 |
| follow-up | 35 | 2010 | 2014 | 2021 |
| down-regulation | 9 | 2009 | 2014 | 2020 |
| lung | 28 | 2007 | 2016 | 2022 |
| progression | 28 | 2007 | 2016 | 2022 |
| prognostic-significance | 48 | 2007 | 2017 | 2022 |
| risk-factors | 16 | 2008 | 2017 | 2021 |
| colon-cancer | 135 | 2008 | 2019 | 2022 |
| risk | 58 | 2008 | 2019 | 2021 |
| colorectal-cancer | 257 | 2007 | 2020 | 2021 |
| survival | 160 | 2010 | 2020 | 2021 |
| impact | 90 | 2020 | 2021 | 2022 |
| total mesorectal excision | 46 | 2011 | 2021 | 2022 |
| tumor deposits | 34 | 2020 | 2022 | 2022 |
| guidelines | 29 | 2021 | 2022 | 2022 |
